# Comment on “Postpartum well-being in hemophilia carriers and women with von Willebrand disease”

**DOI:** 10.1016/j.rpth.2026.106638

**Published:** 2026-05-09

**Authors:** Abraham Majluf-Cruz

**Affiliations:** Unidad de Investigacion Medica en Trombosis, Hemostasia y Aterogenesis, Instituto Mexicano del seguro Social, Ciudad de Mexico, Mexico

*Dear Editor:*

Carriers of hemophilia A or B or patients with von Willebrand disease (VWD) are at high risk for postpartum hemorrhage (PPH, ≥500 mL of blood loss within 24 hours postpartum) or severe PPH (≥1000 mL) [1,2], a life-threatening condition. Among the congenital bleeding diseases, deficiencies of factor (F)VIII/FIX and von Willebrand factor, are the most frequent conditions. For example, VWD may affect ∼1% of the worldwide population, including women and men from all races, both children and adults, who can be variably affected. Pregnant women have an improved hemostatic capacity due to natural procoagulant changes, a mechanism that helps to reduce the risk of bleeding during delivery [[3], [4], [5]]. However, women with VWD and carriers of hemophilia A or B have a significant risk of pregnancy-related bleeding episodes despite the expected hemostatic compensatory response; as a result, a considerable proportion of these patients will experience postpartum or postcesarean section PPH. Although recently challenged [6], one may expect that enhanced hemostatic treatment improves the PPH scenario, but in the real world, unfortunately, both the risk and prognosis do not get better if the diagnosis is unknown because a specific treatment cannot be indicated [7]. Therefore, carriers of hemophilia or patients with VWD truly have a significant risk for PPH.

In current medical practice, besides achieving physical success, obtaining satisfactory results regarding psychological and social issues is also crucial; however, these goals are often overlooked in clinical assessments. Indeed, advances in hemophilia treatment such as prophylactic factor replacement, extended half-life products or bypassing agents are associated with effective results in terms of reduced bleeding, enhanced joint health and function, and improved patient satisfaction [[8], [9], [10], [11]]. However, despite these advances, patients with hemorrhagic disorders are frequently exposed to disparities in medical attention: gender, access to treatment, adherence issues, presence of inhibitors, heterogeneity based on patient backgrounds, and inconsistent psychosocial support. Under specific circumstances such as pregnancy, these factors may induce depression, anxiety, or social withdrawal, which are not adequately addressed by current clinical metrics [10,12,13], affecting patient-reported outcomes [[14], [15], [16]]. Indeed, despite progress in hemophilia care, the relationship between clinical outcomes and quality of life (QoL), remains insufficiently understood because clinical improvement is not always related to well-being. As such, future research and clinical practice must adopt strategies in which all QoL aspects should be considered. Therefore, studies exploring not only the physical but also the psychological and social issues that affect patients with hemorrhagic disorders are very welcome.

PPH, one of the most important postpartum complications, is responsible for almost half of all maternal deaths in underdeveloped countries. PPH represents a threat for women with a congenital bleeding disorder but, because of its high incidence, patients with VWD have the highest risk of experiencing bleeding complications. Of course, once informed about their bleeding status, it is easy to understand why they become anxious regarding pregnancy, a fact even worse if they have already experienced a PPH. Concerns regarding the health status of their children are also understandable in these patients. Occasionally, anxiety may reach elevated levels, leading to a depressive status. These facts may not be epidemiologically relevant; however, they are strongly associated with decreased QoL. Today, QoL is a forgotten issue in most medical areas, not to mention in rare disorders. Study of QoL in congenital bleeding disorders, specifically in women with PPH, has been an almost abandoned field.

In this issue of RPTH, de Vaan et al. [17] inform their findings regarding important QoL aspects in adult postpartum hemophilia carriers and patients with VWD [17]. In this prospective research, part of the PRIDES study, obstetric and gynecologic data were gathered from 2 cohorts separated in time. A major difference between the cohorts was enhanced hemostatic management because von Willebrand factor/FVIII/FIX levels were raised from ≤100 IU/dL up to ≥150 IU/dL. The main objectives were to evaluate their postpartum QoL, childbirth satisfaction, and experience at weeks 1 and 6 postpartum compared with the general Dutch population. QoL was assessed using 3 accepted and validated patient-reported outcome scales. After analyzing data from 85 hemophilia carriers and 81 patients with VWD, authors found that patients experienced good QoL and childbirth satisfaction in the postpartum period although QoL was lower at weeks 1 and 6 compared with the general population but childbirth satisfaction was significantly higher vs the general population. Authors speculate that the last point was due to better management of expectations in the patients.

Important considerations must be raised regarding the findings of this study. First, due to the nature and consequent methodological limitations of this research in which patients from 2 different cohorts were compared, the results should be taken carefully. However, the study shows that increasing QoL in these patients is possible when better treatment and medical attention are provided. Second, QoL scales are essential for evaluating the impact of hemophilia and its treatment on physical, psychological, and social well-being [[18], [19], [20]]. The scales used in this research have been validated, and although no perfect tools for measurement of QoL exist, we may assume that the results really reflect an improved QoL of these patients over time. Third, as mentioned earlier, a recently released article that included only hemophilia carriers (also derived from the PRIDES study) found that enhanced peripartum hemostatic management did not decrease PPH incidence. The results of these 2 subanalyses, derived from the same database, deserve attention because of the apparent divorce between them. How to explain the fact that enhanced hemostatic treatment is associated with a better QoL after PPH without an improved incidence of this complication? Inclusion of hemophilia carriers but not patients with VWD may, at least partially, explain these divergent results. The, perhaps, improved hemostatic treatment plus other nonhemostatic strategies are truly responsible for the positive findings observed in QoL. Indeed, current access to better prenatal care including iron or folic acid supplementation to avoid anemia, improvements in analgesia or anesthesia or antibiotics in association with better general medical care and access to information about the disease, may translate into a more confident mother and better well-being. Therefore, although more replacement treatment does not decrease PPH incidence, when associated with improved global medical, social, and psychological support, it may result in better QoL.

In conclusion, results from the study by de Vaan et al. [17] are hopeful. They showed that, with the passing of time, improvements in global medical care made it possible to increase the QoL of patients with congenital hemorrhagic disorders in a terrible clinical scenario such as PPH.
